# Supplementary figures and images for: Terrorism group prediction using feature combination and BiGRU with self-attention mechanism
Source: PeerJ Comput Sci. 2024 Sep 20;10:e2252. doi: 10.7717/peerj-cs.2252 (PMC11419613; doi:10.7717/peerj-cs.2252)

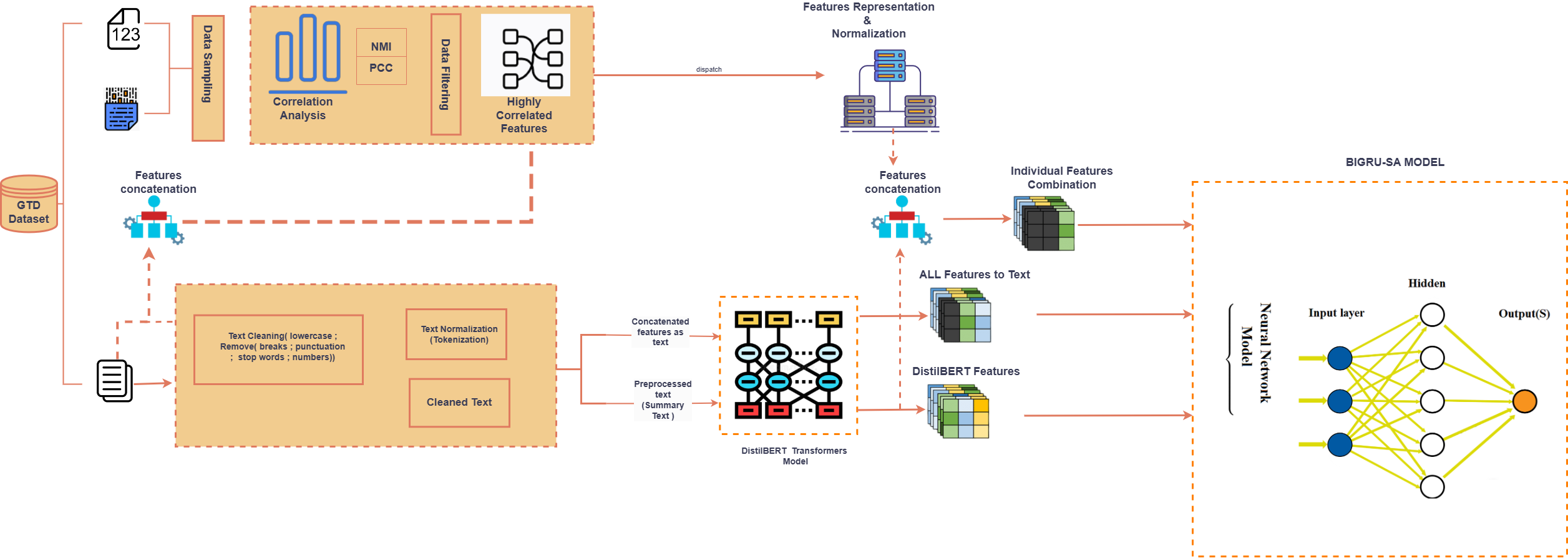

Supplement: Supplemental Information 4 — This flowchart illustrates the steps involved in the proposed framework for classifying and predicting terrorist groups responsible for attacks. The framework utilizes bidirectional recurrent units and self-attention mechanisms (BiGRU-SA), combining textual features extracted by DistilBERT with related features that show a high correlation with terrorist organization activities. The data imbalance is addressed using the Synthetic Minority Over-sampling Technique with Tomek links (SMOTE-T). Source: © diagrams.net (formerly draw.io) [file peerj-cs-10-2252-s004.png]
